# Post-exposure rate of tuberculosis infection among health care workers measured with tuberculin skin test conversion after unprotected exposure to patients with pulmonary tuberculosis: 6-year experience in an Italian teaching hospital

**DOI:** 10.1186/1471-2334-14-324

**Published:** 2014-06-12

**Authors:** Alba Muzzi, Elena Seminari, Tiziana Feletti, Luigia Scudeller, Piero Marone, Carmine Tinelli, Lorenzo Minoli, Carlo Marena, Patrizia Mangiarotti, Maurizio Strosselli

**Affiliations:** 1 Direzione Medica di Presidio, Fondazione IRCCS Policlinico San Matteo, viale Camillo Golgi 19, Pavia 27100, Italy; 2 Clinica Malattie Infettive, Fondazione IRCCS Policlinico San Matteo, viale Camillo Golgi 19, Pavia 27100, Italy; 3 Medicina del Lavoro, Fondazione IRCCS Policlinico San Matteo, viale Camillo Golgi 19, Pavia 27100, Italy; 4 Biometric Unit, Fondazione IRCCS Policlinico San Matteo, viale Camillo Golgi 19, Pavia 27100, Italy; 5 SC Virologia e Microbiologia, Fondazione IRCCS Policlinico San Matteo, viale Camillo Golgi 19, Pavia 27100, Italy; 6 Malattie Apparato Respiratorio, Fondazione IRCCS Policlinico San Matteo, viale Camillo Golgi 19, Pavia 27100, Italy

**Keywords:** Tuberculosis, Occupational TB, Infection control

## Abstract

**Background:**

This study assesses the risk of LTBI at our Hospital among HCWs who have been exposed to TB patients with a delayed diagnosis and respiratory protection measures were not implemented.

**Methods:**

All HCWs exposed to a patient with cultural confirmed pulmonary TB and respiratory protection measures were not implemented were included. Data on TST results performed in the past (defined as T0) were recorded. TST was performed twice: first, immediately after exposure to an index patient (T1) and three months later (T2). The period of time between T0 and T1 was used to calculate he annual rate of tuberculosis infection (ARTI), while le period of time between T1 and T2 was used to calculate the post exposure annual rate of tuberculosis infection (PEARTI).

**Results:**

Fourteen index patients were admitted; sputum smear was positive in 7 (58.3%), 4 (28.6%) were non-Italian born patients. 388 HCWs were exposed to index patients, a median of 27 (12-39) HCW per each index patient. One hundred eighty (46.4%) HCWs received BCG in the past. One hundred twenty two HCWs (31%) were TST positive at a previous routine screening and not evaluated in this subset. Among the remaining 255 HCWs with negative TST test in the past, TST at T1 was positive in 11 (4.3%). ARTI was 1.6 (95% CI 0.9-2.9) per 100 PY. TST at T2 was positive in 9 (3.7%) HCWs, that were TST negative at T1. PEARTI was 26 (95% CI 13.6-50) per 100 PY. At univariate analysis, older age was associated with post exposure latent tuberculosis infection (HR 1.12; 95% CI 1.03-1.22, p=0.01).

**Conclusions:**

PEARTI was considerably higher among HCWs exposed to index patients than ARTI. These data underscore the overwhelming importance of performing a rapid diagnosis, as well as implementing adequate respiratory protection measures when TB is suspected.

## Background

*M. tuberculosis* is carried by airborne particles, called droplet nuclei, that can be generated when patients with pulmonary or laryngeal TB cough, sneeze or shout. Infection occurs when a susceptible person inhales droplet nuclei containing *M. tuberculosis* that reach the alveoli through the upper respiratory tract and bronchi and correlates with exposition to infectious patients [1].

Tuberculosis has long been recognized as an important hazard for HCWs [2-4], and the risk of latent TB infection is four to six fold greater for those exposed than unexposed to patients with TB [5,6] and increases proportionally with the number of TB admissions in each hospital. The risk is low for fewer than ten TB admission annually per 100 HCWs (less than 0.2 percent), and high (between 1.7 to 3.9 percent) when the ratio of HCWs to the annual number of TB admissions increases [4]. Implementing infection control measures among HCWs decreases the rate of TB transmission [7]. The risk of acquiring TB depends on the level of infectivity of the TB patient, the duration and proximity of the contact, and susceptibility of the subject at risk [1,8]. In hospitals where infection control measures are applied, ARTI decreases [7] or remains low, despite a moderate number of TB admissions [9,10]. In a recent review, ARTI in HCWs varies between 0.2 and 12% in high income Countries [11].

A delay in TB diagnosis is associated with inadequate implementation of respiratory protection measures and represents one of the main risk factors for TB spread the risk of latent tuberculosis infection [3,11,12]. Health care delay, defined as the time from the first consultation with an health provider to the initiation of treatment represents the leading cause of HCW’s unprotected exposure, and is mainly due to difficulties in the diagnosis of pulmonary TB [3,13]. Nosocomial transmission of TB infection has been described in HCWs exposed to patients with delayed diagnosis without respiratory [14,15].

This study assesses the risk of LTBI among HCWs who have been exposed to TB patients with a delayed diagnosis and respiratory protection measures were not implemented at our Hospital.

## Methods

All HCWs exposed to a patient with cultural confirmed pulmonary TB and respiratory protection measures were not implemented were included in the present study.

Data were collected from 1st January 2006 to 31st December 2012. Respiratory protection measures available in our Institution include FFP2 and FFP3 masks for HCWs, negative pressure isolation rooms with 6–12 air changes per hour and ventilation system with high efficiency particulate air (HEPA) filter for patients, and use of surgical masks for patients during any movement to other departments; all these measures are implemented with either known or suspected patients with pulmonary TB.

The patients with cultural confirmed pulmonary TB and delayed diagnosis are defined hereinafter as index patients.

To identify contacts, defined as anyone who was in contact with any index case without respiratory protection measures (i.e. all HCWs who entered the patient room in the ward, those who assisted the patients in the other departments such as casualty, radiology or endoscopic procedure ward), epidemiological investigations were conducted in each ward where the index patient was admitted by the infection control team. All HCWs with a history of contact with an index patient were screened with the TST. Data on TST results performed in the past (mostly at hiring, defined as T0) were recorded. Any HCW with a previous documented positive TST result (in the majority of cases TST was done at hiring) (induration >5 mm considered reactive) or previous TB treatment was not eligible to participate in the present study. TST was performed twice: first, immediately after exposure to an index patient (T1) and second, three months later (T2) in all HCWs who were TST negative at T1.

Therefore the period of time between T0 and T1 was used to calculate he annual rate of tuberculosis infection (ARTI), while le period of time between T1 and T2 was used to calculate the post exposure annual rate of tuberculosis infection (PEARTI).

TST was performed by intradermal injection of 0.1 ml of purified protein containing 5TU (Tuber test, Sanofi Pasteur) and read 72 hours later (known as the Mantoux technique). TST conversion was defined as an induration ≥10 mm in a subject with a previous negative TST [16]. The CDC rule is used to read the Mauntoux test (a pen is used to mark the edges of the induration and a small, plastic, flexible ruler marked in millimeters is used to measure the test) (http://www.cdc.gov/tb).

The study was approved by local Ethics Committee of the Fondazione IRCCS Policlinico San Matteo Hospital. Patients and children’s parents were asked to sign a consent form.

Data on TB patients admitted to this Hospital during the study period were drawn from hospital discharge charts. Chest x-ray on admission was classified as typical for TB if apical fibronodular disease or noncalcified nodules, any mass or pleural disease, parenchymal disease with or without cavities were observed or atypical if normal or minor findings were observed [17].

### Analysis

Continuous variables were described by median values and 1–3 quartiles (interquartile range IQR) and categorical variables by percentages. The Chi-square test was applied to study any association between categorical variables. ARTI and 95% CI were calculated per 100 person-years (date from the first negative result until conversion), assuming that the likelihood of being infected with *M tuberculosis* remained constant throughout the follow up interval [18]. In a fixed period, i.e. one year, one can calculate a conversion rate by dividing the number of conversions among HCWs in the setting (numerator) by the number of HCWs who received tests over the same period (denominator) multiplied by 100. This is also called “incidence risk” or “cumulative incidence”. As there was not a fixed period but follow up time varies among HCWs, data are reported as incidence as number of HCWs who TST converted for 100 person/year. This is also called “incidence rate” or “incidence density”.

PEARTI and 95% CI were calculated per 100 person-years considering date from unprotected exposure to an index patient until TST conversion. Chi square test for trend was utilized to evaluate the TB admission rate throughout the period, assuming that the risk of HCWs to contract TB has been constant during the period if the trend had remained stable.

Poisson regression was applied to the univariate analysis to evaluate the association between TST conversion (dependent variable) at T1 or T2 with age, gender, profession, time since hire, history of Bacillus Calmette-Guèrin (BCG) vaccination of HCWs and positive sputum smear of the index patient.

Data analysis was performed with the STATA statistical package (release 13).

## Results

Fourteen index patients were admitted to a 900 bed teaching Hospital; 11 were males, age was 51 years (IQR 45–54), 4 (28.6%) were non Italian born patients, including a 6 month old boy born in Ukraine and three adults born respectively in Senegal, Romania and China.

Time from admission to TB diagnosis with application of airborne precautions was in median four days (2–19). Sputum smear was positive in seven (58.3%) patients. Sputum culture was positive in all patients (2 strains were resistant to streptomycin and 1 strain was resistant to isoniazid). Two patients also had extra pulmonary involvement (1 had hepatic abscesses and 1 had both central nervous system and renal infections).

Index patient comorbidities included cirrhosis in two patients, advanced cancer in two patients, chronic kidney disease in two patients, HIV infection in one patient and diabetes in one patient.

Chest x-ray showed atypical results, mainly represented by interstitial thickness, in 6 patients; homogeneous consolidation with or without cavitary lesions were seen in 8. Patients with comorbidities had more frequently an atypical chest x-ray (p = 0.02). Among patients born in Italy, 7 out of 10 had comorbidities that might have jeopardized the chest x-ray findings.

Index patients were admitted to the Intensive Care Unit (4 patients), Otolaryngology Unit (1 patient), General Surgery Unit (3 patients), 3 Internal Medicine (3 patients), Palliative Care Unit (2 patients) and Pediatric Unit (1 patient).

Concomitantly, 326 patients with pulmonary TB were admitted to our Hospital, for whom required respiratory protection procedures were implemented. The trend of TB admission was stable over the period (p = 0.8).

**Figure 1:**
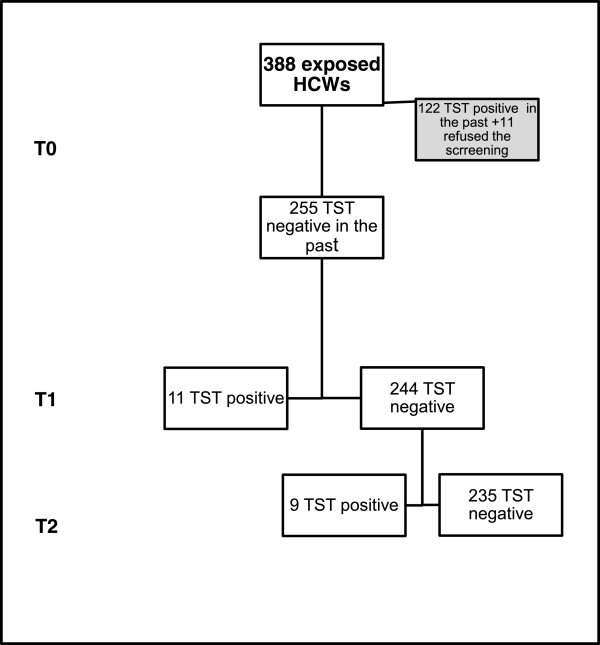
**Flow chart summarizing HCW screening procedures.** T0: TST result in the past (mostly at hiring), T1: TST result at exposure, T2: TST result 3 months after exposure.

388 HCWs were exposed to index patients (Table 1 and Figure 1), a median of 27 (12–39) HCW per each index patient; 122 (31%) were males, age was 40 years (32–47); time from hire was 10.5 years (3.6-18.7); 255 were employed as nurses, 120 as physicians, 13 with other occupations. The breakdown per ward is as follows: 175 (45.1%) medicine, 81 (20.9%) surgery, 85 (21.9%) ICU, 20 (5.2%) radiology, 9 (2.3%) emergency, 18 (4.6%) other. 180 (46.4%) HCWs had received BCG in the past, as self reported.

**Table 1:** Epidemiologic characteristics of exposed HCWs

	**Total exposed HCWs**	**HCWs TST neg in the past. Included in analysis**	**HCWs TST pos in the past. Not included in analysis**
Total n patients	388	255	122 + 11°
TST positive at hire (%)	122 (31)	0	122
Male/female	122/246	82/173	40/93
Age at hiring (years)	40 (32–47)	42 (34–48)	35 (30–42)
Time since hire (years)	10.5 (3.6-18.7)	8.7 (2.6-16)	15 (8.3-20.6)
Profession (%)			
Nurse	255 (65.7%)	158 (62)	97 (73)
Physician	120 (30.9%)	86 (33.7)	34 (25.6)
Other	13 (3.4%)	11 (4.3)	2 (1.5)
BCG in the past (%)	180 (46.4)	75 (41.7)	105 (58.3)
Ward of admission of cases (%)			
Medicine	175 (45.1)	125 (49)	50 (37.6)
Surgery	81 (20.9)	43 (16.9)	38 (28.6)
ICU	85 (21.9)	54 (21.2)	31 (23.3)
Radiology	20 (5.2)	13 (5.1)	7 (5.3)
Emergency	9 (2.3)	7 (2.8)	2 (1.5)
Other	18 (4.6)	13 (5.1)	5 (3.8)

°HCWs who refused to participate in the screening program.

One hundred twenty two HCWs (31%) were TST reactive (induration >5 mm), at a previous screening in the past and therefore TST was not evaluated in this subset, to reduce the risk of boosting effect or false positive test after BCG vaccination. Eleven (4.1%) HCWs refused to participate in the screening program.

Analysis were done using the remaining 255 HCWs with negative TST test in the past, TST at T1 was positive in 11 (4.3%). ARTI was 1.6 (95% CI 0.9-2.9) per 100 person-years. All HCWs who resulted TST negative at T1 were evaluated with TST at T2. TST at T2 was positive in 9 (3.7%) HCWs (Table 2), that were TST negative at T1 (6 nurses and 3 physicians). PEARTI was 26 (95% CI 13.6-50) per 100 person-years. At univariate analysis (Table 3), older age was associated with PEARTI (HR 1.12; 95% CI 1.03-1.22, p = 0.01). No association was observed between any variables analyzed and T1 TST conversion.

**Table 2:** Characteristics of HCWs with TST conversion at T2

**Occupation**	**Year of birth**	**BCG vaccination status (year vaccination)**	**Year of contact with TB patient**	**Ward**	**Year of hiring**
Nurse	1965	Yes (1997)	2006	medicine	1997
Nurse	1972	Yes (1994)	2012	ICU	1997
Nurse	1092	Yes (1990)	2011	ICU	1994
Physician	1978	no	2011	ICU	2011
Nurse	1957	no	2009	medicine	2006
Nurse	1973	Yes (2002)	2008	ICU	2011
Nurse	1964	Yes (1980)	2009	medicine	1990
Physician	1956	Yes (1976)	2009	medicine	1990
Nurse	1955	no	2012	surgery	1982

**Table 3:** Risk factors associated to PEARTI (Univariate analysis)

	**N events**	**Person years**	**Rate (per 100 person years)**	**Hazard ratio 95% confidence interval**	**p**
Age (for each year increase)	9	NA	NA	HR 1.12; 95% CI 1.03-1.22	0.01
Age at hire (for each year increase)	9	NA	NA	HR 1.008; 95% CI 0.93-1.09	0.83
Time since hire (for each year increase)	9	NA	NA	HR 1.06; 95% CI 0.99-1.13	0.08
Gender (male vs female)	F 6	24.5	32.7	HR 0.29; 95% CI 0.04-2.35	0.25
	M 3	10	10.3		
Profession (physician vs nurse)	Nurse 7	23.8	33.64	HR 0.45; 95% CI 0.09-2.17	0.32
	Physician 2	11.8	16.9		
Sputum smear of the index case (positive vs negative)*	Negative 6	14.7	20.5	HR 1.59; 95% CI 0.04-6.3	0.58
	Positive 3	19.6	30.7		
History of BCG (positive vs negative)	Negative 3	19.7	15.3	HR 2.84; 95% CI 0.71-11.34	0.14
	Positive 6	14.6	41.25		

*The sputum smear result of the index case who was in contact with HCWs tested positive at T2.

## Discussion

The delay in TB diagnosis in one of the main risk factors for TB contagiousness as it is associated with inadequate implementation of respiratory protection measures and represents one of the main risk factors for TB spread [11,12,14,15]. It can be divided into “patient delay” that is defined as the time from onset of TB symptoms to the first consultation with a health provider and “health care delay” that is defined as the time from the first consultation with a health provider to the initiation of treatment and “total delay” defined as the time from onset of TB symptoms to the initiation of treatment [19].

Factors associated with delayed TB diagnosis are heterogeneous and include demographic (older age, female) and socioeconomic factors (low income, low educational level) and clinical characteristics (negative sputum smear, smoking, sexually transmitted disease as comorbidity, less severe and atypical symptoms) [12]. The ARTI of HCWS at our Institution during the study period was comparable to ARTI reported in the medical literature for high income countries with a moderate number of TB admissions, and it is estimated at around 1% (ranging from 0.2% to 12%) [11]. The risk of LTBI dramatically increases when airborne protection procedures are not in place in the presence of active pulmonary TB, mainly due to delayed diagnosis, as demonstrated by the increased value of PEARTI compared with ARTI. It is interesting that the majority of index patients that elude a prompt diagnosis were Italian born patients who had a chest-x ray that was negative for cavitary lesions and was not specifically indicative of TB disease. Due to the combination of epidemiological and radiological issues, the suspicion of clinicians was low and therefore the patients were initially misdiagnosed.

In a recent paper published by Italian authors and conducted in the Emilia Romagna region, where TB epidemiology is similar to the Lombardy region, where our hospital is located, the median health care delay in TB diagnosis was significantly higher for Italian as compared with foreign born patients (60 vs 18 days) [19]. It seems that Italian clinicians in general, show a higher suspicion index for migrants than for Italian born patients. Italy has a low incidence of TB with 7.6/100.000 patients reported in 2008 [20]. In some industrialized areas, however, as in Lombardy the incidence reported was 13/100,000 population, and TB is increasingly associated with specific population subgroups: immigrants from countries with a high endemic infection, elderly Italian natives and the homeless. The majority of TB patients in Italy are foreigners aged 25–34 and, less frequently, older (>65 year) Italian citizens [21].

Among variables analyzed (gender, occupation, time since hire, history of BCG vaccination, positive sputum smear of index patient), only older age was associated with PEARTI. Age is a known risk factor for LTBI infection among HCWs [22,23], possibly because it is associated with a longer period of direct contact with patients. This observation might suggest a limitation of the study, as PEARTI was measured as TST conversion three months after the exposure following a first negative TST result. This may represent a “booster effect”, an increase in the size of the induration produced by the Mantoux test due to the repetition of the test and secondary to the stimulation of cellular immunity in the absence of new *M. tuberculosis* infection [24]. However, also taking into account the possibility of a booster effect of about 15% [25], these data underscore the high infectivity of a misdiagnosed TB patient admitted to a hospital. Moreover, in the univariate analysis the history of BCG vaccination was not associated with TST conversion neither at T1 nor at T2. It is important that clinicians always maintain a high level of suspicion associated with knowledge of local TB epidemiology in order to avoid delayed diagnosis and TB spread among HCWs and other contacts (relatives, friends, colleagues etc.). Our data of TST conversion after unprotected exposure to TB patients are consistent with those recently reported by Harris and coworkers [14]. The exposure to an unrecognized Tb patient had as a consequence a TST conversion for 8% of staff members of a long term care facility, and two secondary cases of pulmonary TB among patients [14]. These data underscore the infectiveness of pulmonary TB and the necessity of a the importance of considering TB when evaluating patients with pulmonary symptoms, particularly when symptoms persist or recur regardless antibiotic treatment [14].

Other limitations of the study are represented by the possibility of intra/inter reader errors in evaluating TST results, but as we considered as positive an induration equal or above 10 mm, and as the team has standardized methods, the possibility of different interpretation of the same TST result should be exiguous. Moreover, a HCWs could have been infected with *M.tuberculosis* outside the hospital, in the same timeframe. It not possible to rule out this hypothesis, but considering the epidemiology of tuberculosis in our region and the value of ARTI, the probability is low.

## Conclusion

In conclusion PEARTI was considerably higher among HCWs exposed to patients than ARTI, even taking into account the possibility of a booster effect. These data underscore the overwhelming importance of performing a rapid diagnosis, as well as maintaining a high level of suspicion in order to implement adequate protective respiratory measures.

## Abbreviations

ARTI: Annual rate of tuberculosis infection; BCG: Bacillus Calmette–Guérin; HCW: Health care worker; HEPA: Filters high efficiency particulate air filter; IQR: Interquartile range; LTBI: Latent tuberculosis infection; PEARTI: Post exposure annual rate of tuberculosis infection; TB: Tuberculosis; TST: Tuberculin skin test.

## Competing interests

The authors have no competing interests to declare.

## Authors’ contributions

AM, ES, LS, CT designed the study and drafted the manuscript. CM and AM were responsible for the management of exposed HCWs. TF, MS, PiM and PaM were involved in the epidemiological investigation and clinical follow up of HCWs exposed to index patients. ES and LM were in charge of index patients. LS and CT performed the statistical analysis. All authors reviewed the manuscript and approved the final version.

## Pre-publication history

The pre-publication history for this paper can be accessed here:

http://www.biomedcentral.com/1471-2334/14/324/prepub
